# A Novel Graphite Fluoride/Bioactive Glass-containing Orthodontic Primer with Antibacterial and Remineralization Properties: An In-vitro Study

**DOI:** 10.3290/j.jad.b5793278

**Published:** 2024-10-21

**Authors:** Afaf H. Hussein, Yassir A. Yassir

**Affiliations:** a Postgraduate Orthodontic Student, Department of Orthodontics, College of Dentistry, University of Baghdad, Iraq. Idea, experimental design, performed the experiments in partial fulfillment of requirements for a PhD degree, wrote the manuscript.; b Professor, Department of Orthodontics, College of Dentistry, University of Baghdad, Iraq; School of Dentistry, University of Dundee, UK. Idea, experimental design, proofread the manuscript, consulted on and performed a statistical evaluation.

**Keywords:** antibacterial, bioactive glass, graphite fluoride, orthodontic primer, remineralization, white spot lesion

## Abstract

**Purpose:**

This study aimed to develop a novel orthodontic primer that incorporated graphite fluoride (GF) and Bioactive glass (BAG) and to investigate its cell viability, bonding strength, and enamel damage, as well as its antibacterial and remineralization properties.

**Materials and Methods:**

Nine groups were prepared by adding different concentrations of GF (1, 2, and 4 wt.%) and BAG (1, 3, and 5 wt.%) to Transbond™ XT orthodontic primer. The prepared primers were compared to the control primer in terms of cell viability, shear bond strength (SBS), adhesive remnant index (ARI), enamel damage index (EDI), and antibacterial test. Then, the groups with better antibacterial properties (GFBAG 1-1, GFBAG 4-1, GFBAG 4-3, GFBAG 4-5) were evaluated for the remineralization properties.

**Results:**

All the prepared orthodontic primers with different concentrations of GF/BAG revealed acceptable cell viability levels, with comparable SBS and ARI values to the control primer (p>0.05). Simultaneously, the EDI was reduced, while the antibacterial properties were significantly enhanced when compared to the control group (p<0.05). The result of remineralization properties revealed that the selected groups had significantly higher remineralization ability than the control group; this was most pronounced in the GFBAG 4-3 group.

**Conclusions:**

All the prepared GF/BAG orthodontic primers are biologically safe with adequate SBS, ARI, and EDI values for clinical application with enhanced antibacterial properties. The GFBAG 4-3 experimental primer reveals the best antibacterial and remineralization properties which require further *in-vitro* and *in-vivo* investigations as a preventive measure of white spot lesions.

The development of a white spot lesion (WSLs) is one of the most frequent esthetic and clinical adverse effects of fixed orthodontic therapy.^38^ Fixed orthodontic appliance is usually accompanied by difficulties in maintaining good oral hygiene due to their irregular surfaces, which reduce the efficiency of self-cleansing properties, and increase plaque accumulation and proliferation of cariogenic bacteria. So, the risk of demineralization and WSLs development around the appliance increased with the presence of low pH.^44^ The prevention of WSLs is a multifactorial process based on the concept of demineralization inhibition and/or remineralization enhancement.^34^

Graphite fluoride (GF) is an uprising element in the graphene derivatives family with a fluorine-containing platelet structure. Fluoride inhibits bacterial action by intervening with its metabolic processes, reducing the acid production and subsequent demineralization of the teeth. Furthermore, fluorapatite will be formed making the tooth surface more resistant to acidic conditions.^48^

Bioactive glass (BAG) is a SiO_2_-based material that is usually combined with Na_2_O, CaO, and P_2_O_5_ with a specific number of other modifiers to manage its biocompatibility and characteristics.^5^ BAG is a biomaterial that has attracted attention in recent years. When BAG is incorporated into a material, it acts as a filler in addition to its buffering effect, antibacterial, and ion-resolution properties. BAG releases Na⁺, Ca_2_^+^, and PO_4_^3–^ ions on the moist tooth surface, creating a saturated ion state to transform the calcium phosphate layer into apatite crystal, elevating the pH, in addition to its antibacterial effect.^49^

The improvement of antibacterial and remineralizing properties of orthodontic bonding systems by the addition of biomaterials should be biologically safe for humans while maintaining their mechanical and physical properties. So, evaluation and optimization of the material properties are crucial for the new material development to be suitable for clinical application in a complicated oral environment.^40^ These biomaterials act as fillers and play a vital role in stress absorption by acting as an elastic layer in the adhesive-enamel interface. However, experimental studies need to optimize the distribution and amount of these biomaterials before their clinical application.^1^ Graphene and its derivatives have attracted attention for dental research in recent years due to their chemical and thermal stability with antibacterial properties.^6^ Most of the investigations were carried out on graphene oxide and reduced graphene oxide. The antibacterial properties of graphene can be enhanced by the fluoride-releasing properties of GF with the ability to form fluorapatite, rendering the tooth surface more resistant to demineralization.^62^ However, there is a limited number of studies on the dental application of GF and no previous study on their application in orthodontic primer.

Modifying orthodontic adhesive by the addition of biomaterials has been investigated by many researchers to improve their antibacterial and remineralization properties.^16,17,24, 40,43,48,49,54^ While limited studies have evaluated the enhancement of primer properties by biomaterials.^1,3,18,39,68^ Incorporating these materials into the orthodontic primer rather than adhesive could seem more reasonable since the primer will come in intimate contact with the enamel which is the target area for prophylactic measurements.^1^ Due to the potential effect of GF and BAG in reducing WSLs formation, this study aimed to develop a novel orthodontic primer incorporating GF and BAG. The objectives of this study were to prepare and test GF/BAG-containing orthodontic primer and to determine its effect on cell viability, bracket shear bond strength, adhesive remnant index (ARI), enamel damage index (EDI), antibacterial, and remineralization properties.

The null hypothesis is that “there are no significant differences between orthodontic primer-containing GF/BAG and the control primer in terms of biocompatibility, bonding strength, ARI, EDI, antibacterial, and remineralization effect.”

## Materials and Methods

### Characterization of Nanomaterials

Graphite fluoride powder with 8–10 µm particle size (Nanografi Nanotechnology; Jena, Germany) and Bioactive glass powder 45S with 0.2–500 μm particle size, ≥ 98% purity (Sigma-Aldrich; MA, USA) were purchased. The physical structure of the materials was analyzed using a field emission scanning electron microscope (FESEM) (InspectTM F50, FEI; Hillsboro, USA) at an accelerating voltage of 30 kV under low vacuum operation. The phase of the materials was evaluated by radiographic diffractometer (XRD) (Lab X, XRD-6000, Shimadzu; Kyoto, Japan), scanning was done using continuous scan mode, 5,000–60,000 scan range, two theta drive axis, using Cu as a target with a voltage of 40 kV and current of 30 mA. The chemical compositions were analyzed under a low vacuum by Energy dispersive radiographic spectroscopy (EDX) (Thermo Fisher Scientific; Waltham, USA).

### Preparations of Experimental Primers

A series of experimental primers were prepared by adding different concentrations of GF (1, 2, and 4 wt.%) and BAG (1, 3, and 5 wt.%) to Transbond™ XT orthodontic primer (3M, Uniteck; Monrovia, USA). Precise weighting was achieved using a four-digit weight balance (KERN, ALS220-4N; Balingen, Germany) and added to plain primers by wt.:wt. ratio to develop nine experimental primers, while plain Transbond™ XT orthodontic primer was used as a control (Table 1). The materials’ addition and mixing were performed in a black glass tube to prevent light polymerization of the developed primers. In the mixing procedure, a straight handpiece with a custom-made spatula was used at a different speed for 3 min to achieve adequate homogeneity.^3,4^

**Table 1:** Composition of the control and experimental groups containing GF/BAG

Group	Transbond™ XT primer (wt.%)	GF (wt.%)	BAG (wt.%)
Control	100%	0%	0%
GFBAG 1-1	98%	1%	1%
GFBAG 1-3	96%	1%	3%
GFBAG 1-5	94%	1%	5%
GFBAG 2-1	97%	2%	1%
GFBAG 2-3	95%	2%	3%
GFBAG 2-5	93%	2%	5%
GFBAG 4-1	95%	4%	1%
GFBAG 4-3	93%	4%	3%
GFBAG 4-5	91%	4%	5%

### Cell Viability Assessment

L929 mouse fibroblast cell line (ATCC; VA, USA) was used for assessment of cell viability. Monolayer culture of the cell line was obtained by their cultivation in RPMI 1640 medium (Euroclone; Milano, Italy) containing 10% fetal bovine serum (Cytiva; Marlborough, USA), 100 IU/mL penicillin (Euroclone; Milano, Italy), and 100 µg/ mL streptomycin (Euroclone; Milano, Italy) at 37°C in CO_2_ incubator (GENEX; FL, USA).^42,45^

#### Primer extract preparation

A total of 90 disks of 0.025 ± 0.001 g (nine disks for each of the control and experimental primers) were prepared, disks were prepared by dripping the primer using a micropipette into a custom-made Teflon mold with 5 mm in diameter and 1 mm in thickness.^20^ All the disks were sterilized from both sides using ultraviolet light for 45 min in the laminar flow cabinets (Daihan Labtech; Namyangju, South Korea).^45,51^ The elutes were prepared by immersion of prepared disks (with 0.5497 cm^2^ surface area) in 180 µl RPMI 1640 medium (Euroclone; Milano, Italy) to obtain 3 cm^2^/ml disk surface area to medium volume ratio as recommended by ISO 10993-12 Specification (2012).

The elutes were prepared by conditioning the disks at 37°C for 24, 48, and 72-h immersion periods.^14,15^

#### MTT assay

The cells with a density of 4×10⁴ per well were seeded and cultured for 24 h at 37°C in a 96-well plate; then the cells were cultured with 100 µl of prepared experimental primer extracts with different exposure times, while the untreated culture medium was used as a negative control.^14,41^ After 24 h of incubation, the primer extracts were discarded, replaced by 50 µl of MTT reagent (5 mg/ml; Abcam; Cambridge, UK), and incubated at 37°C for 4 h.^55,60^ Cell viability was evaluated by triplicate measurement of the optical density of formed blue-violet formazan dye by a microplate reader (Bio-Rad; Hercules, CA, USA) at 590 nm. The cell viability was calculated by using the following equation:

Cell viabilities % = (A/ B) × 100

A and B are the optical densities of the tested primer and negative control (the cell without primer disk).^15,50^ All the measurements were performed by a specialized biologist who was blinded to the study groups.

### Shear Bond Strength (SBS)

The sample size for SBS was calculated by G-power software (version 3.1.9.7; Franz Faul, Germany) based on the expected SBS of 25 MPa and standard deviation (SD) of 3.6^39^ using a two-tailed test with a 0.05 alpha level and a power of 80%. A sample of ten teeth for each group was needed to detect 20% difference between the groups, based on the method used by Reis et al.^58^

After obtaining ethical approval from the ethics committee, a total of 100 premolar teeth (extracted for orthodontic purposes) with intact enamel surfaces, free of cracks and caries were selected following microscopical examination (Hamilton, Altay; Roma, Italy) under 10× magnification.^56^ All the teeth were cleaned using a scaler (Pyon2 Ultrasound Piezo scaler, W&H; Bürmoos, Austria) under running water and stored in 1% chloramine-T trihydrate (Sigma-Aldrich; MA, USA) for one week, followed by storage in distilled water.^28^ The teeth were randomly divided and coded by an independent person into ten groups with ten samples for each group and mounted in a standardized procedure to acrylic blocks.^59^ The buccal surfaces of the teeth were prepared for bonding by polishing using a rubber cup and handpiece for 10 s with fluoride-free pumice (Willmann & Pein; Barmstedt, Germany) and conditioning for 30 s with 37% phosphoric acid gel (SDI; California, USA). Then a thin layer of either the control or one of the experimental primers was applied on the etched tooth surface and light-cured with O-Star light curing LED (Woodpecker; Guilin, China) in orthodontic mode (2,700–3,000 mW/cm^2^) for 3 s from the mesial and distal side according to the manufacturer’s instructions. A small amount of adhesive (Transbond™ XT, 3M, Unitek; Monrovia, USA) was applied on the bracket base (DB Orthodontics; Yorkshire, UK) and positioned on the tooth surface with an application of 300 g load (measured by force gauge) to produce a uniform thickness of adhesive.^31^ After curing, the bonded teeth were stored in distilled water in an incubator (Faithful Instrument; Huanghua, China) at 37°C for 24 h.^10^ The universal testing machine (Instron Laryee; Beijing, China) at 0.5 mm/min crosshead speed was used to apply occluso-gingival force from the chisel to the enamel-bracket interface until bracket debonding to measure the SBS in Newtons then converted to megapascal (MPa) by dividing the SBS value to the bracket base surface area (9.8172 mm) as supplied by the manufacturer.^47^

### Adhesive Remnant Index (ARI) and Enamel Damage Index (EDI)

After debonding, the amount of remaining adhesive on the tooth surface was evaluated by stereomicroscope (Hamilton, Altay; Roma, Italy) under 10× magnification using the ARI with the following scores: 0, no adhesive left on the tooth surface;1, less than half of the adhesive left on the tooth surface; 2, more than half of the adhesive left on the tooth surface; and 3, all the adhesives left on the tooth surface.^11^

Enamel surface damage was evaluated after the removal of the residual adhesive by 12-bladed tungsten carbide bur at 20,000 rpm using a low-speed handpiece and polishing with a rubber cup and pumice (Willmann & Pein; Barmstedt, Germany).^12^ The teeth were examined by stereomicroscope (Hamilton, Altay; Roma, Italy) under 40× magnification to evaluate EDI with the following scores: 0, no cracks or tear-outs were seen on the enamel surface; 1, only a crack was seen on the enamel surface; 2, only tear-out seen on the enamel surface; and 3, both crack and tear-out were seen on the enamel surface.^36^

### Antibacterial Test

The antibacterial properties were evaluated against *Streptococcus mutans* (the main microorganism responsible for WSLs development) using agar diffusion methods on Mueller Hinton agar (HIMEDIA; Mumbai, India). The tested samples were prepared by the addition and curing of 10 µl of the control or one of the experimental primers to standardized sterile (6 mm in diameter) Whatman no. 1 filter paper (GE Healthcare Co.; Buckinghamshire, UK) disks (ten for each group) and a filter paper disk saturated with distilled water was used as a negative control.^30^ Fifty µl of the prepared standardized bacterial suspension with 0.5 McFarland scale was placed on the surface of Mueller Hinton agar (HIMEDIA; Mumbai, India) and spread with a sterile swab. Then the prepared primer disks were gently applied and adapted on the agar surface with sterilized tweezers and incubated at 37°C for 24 h before measuring the bacterial growth inhibition zone in millimeters around each prepared disk.^57^

### Evaluation of WSLs Development and Mineral Component of Enamel Surface

According to the result of the antibacterial test, four groups with higher antibacterial properties (GFBAG 1-1, GFBAG 4-1, GFBAG 4-3, GFBAG 4-5) were selected to evaluate their ability to prevent WSLs development after pH cycling.

#### Sample preparation and pH cycling

Fifty premolar teeth with intact enamel surface, free of cracks and WSLs were selected and randomly divided and coded into five groups of 10 (one control group, and four experimental groups). The root of each tooth was removed by horizontal sectioning below the cementoenamel junction,^22,63^ and the crowns were embedded separately in auto-polymerized acrylic blocks keeping the buccal surface exposed parallel to the mold base.^23,67^ The tooth surface is polished using a rubber cup and handpiece with fluoride-free pumice (Willmann & Pein; Barmstedt, Germany), dried, and covered with adhesive tape, leaving a 3.5×3.5 mm window on the tooth surface exposed at the bonding site (resembling the bracket size). The exposed tooth surface was etched with 37% phosphoric acid (SDI; California, USA), then the tape was removed, and the bonding procedure was completed as in the SBS test, using one of the experimental or control primers.^52^ All the bonded teeth were incubated for 24 h in distilled water before pH cycling.^40,48^ The Featherstone pH cycling model was used by immersion of the samples in a remineralizing solution at 37°C for 18 h and then immersing in a demineralizing solution at 37°C for 6 h with rinsing by distilled water for 1 min between the two solutions. This cycle was repeated for 2 weeks, and the solutions were replaced weekly.^9^

#### Laser fluorescence measurements

Fifty premolar teeth were selected (ten for each group) for laser fluorescence measurements by DIAGNOdent pen (KaVo, Dental Excellence; Biberach, Germany) after calibration with the ceramic standard disc.^29^ On the other hand, the intraexaminer reproducibility of the measurements of the investigated area was repeated with a 4-week interval and assessed by calculation of interclass correlation coefficient (ICC) [0.96 (95% CI 0.84–0.99)] which reflects a high level of reliability.

The initial fluorescent measurement of each tooth was performed after bracket bonding. The teeth surfaces were dried for 5 s, and the measurements were undertaken 1 mm away from the bracket base on the occlusal, gingival, and proximal surfaces by the perpendicular placement of the tip.^46,65^ The highest fluorescence value was recorded, the measurement was repeated three times, and their mean value was recorded as (F1). The same measurements were repeated after pH cycling and recorded as (F2). The difference between the two records (ΔF= F1–F2) was used to represent the difference in the fluorescent value after pH cycling in the control and experimental groups.^19^

#### Assessment of mineral contents of enamel topography

Chemical elemental analysis of the enamel surface of the control and experimental groups (n = 5 each) was performed by using EDX (Thermo Fisher Scientific; Waltham, USA).^7,63^ The weight percentage of calcium and phosphorus were assessed 1 mm away from the gingival bracket base (the area with the higher risk of WSLs development) immediately after the bracket bonding (M1) and after the pH cycling (M2). Three values were measured, and their mean was recorded.^32,33^ The difference between the two measurements (ΔM = M2 – M1) gave information about the changes in the weight percentage of calcium and phosphorus after pH cycling.^22,64^

### Statistical Analysis

The collected data was analyzed using the Statistical Package for Social Science software computing program (SPSS, version 26, SPSS; Chicago, IL, USA). Data were screened for normal distribution and homogeneity using the Shapiro–Wilk and Levene’s tests, respectively. Descriptive statistics including mean, median, minimum, maximum, interquartile range, and SD were used. One-way analysis of variance (ANOVA) and Tukey HSD post hoc multi-comparison tests were performed for continuous data with normal distribution. Welch and Games–Howell tests were used when there was a concern about the homogeneity of data. While Kruskal–Wallis and pairwise comparisons were conducted to compare the categorical data. All statistical analyses were considered significant at a level of p <0.05.

The sample size was determined using convenience sampling, which was based on previous studies for cell viability, antibacterial test,^25^ laser fluorescence measurements,^46^ and EDX.^70^

## Results

### Characterization of GF and BAG

**Fig 1:**
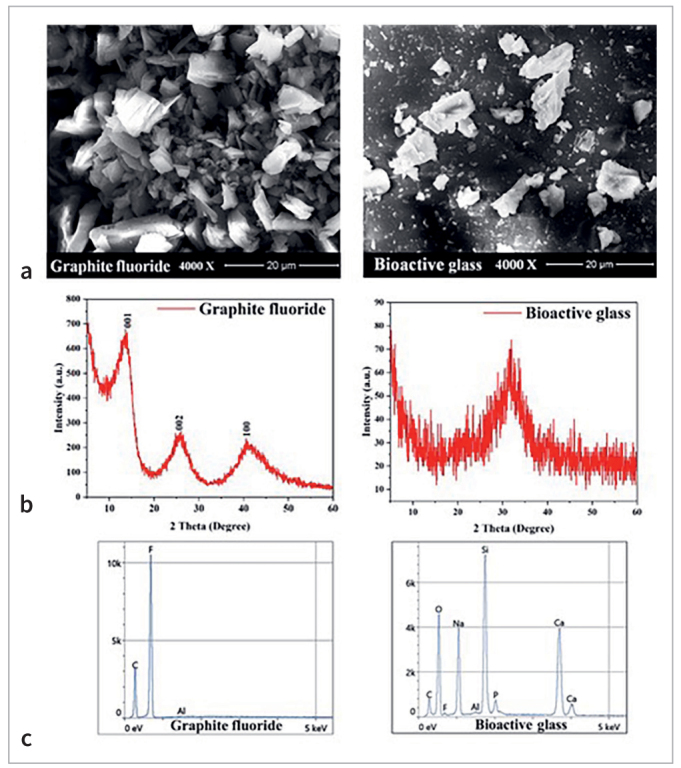
Characterization of GF and BAG; a: FESEM, b: XRD, c: EDX.

The FESEM images (Fig 1a) of GF show a flake-like, well-defined closely stacked layer, while BAG has polygonal particles with irregular morphology. The XRD diffractograms of GF (Fig 1b) show a crystalline structure with the most characteristic peaks observed at 2θ = 14.1, 25.4, and 42.5. The strongest peak (001) indicates the presence of a hexagonal structure while the broad peaks refer to poor ordering in their stacking direction. The average crystal size was calculated using the Scherrer equation, which was approximately 9.6 nm. No crystalline peak was observed in the XRD pattern of BAG with a halo centered around 2θ = 30 indicating the presence of an amorphous structure. The EDX analysis (Fig 1c) shows that GF is mainly composed of C (32.2 wt.%) and F (67.7 wt.%). The EDX result of BAG shows that O (43.0 wt.%), Na (16.7 wt.%), Si (15.6 wt.%), C (11 wt.%), and Ca (10.7wt.%) are the most predominant elements.

### Cell Viability Assessment

**Fig 2:**
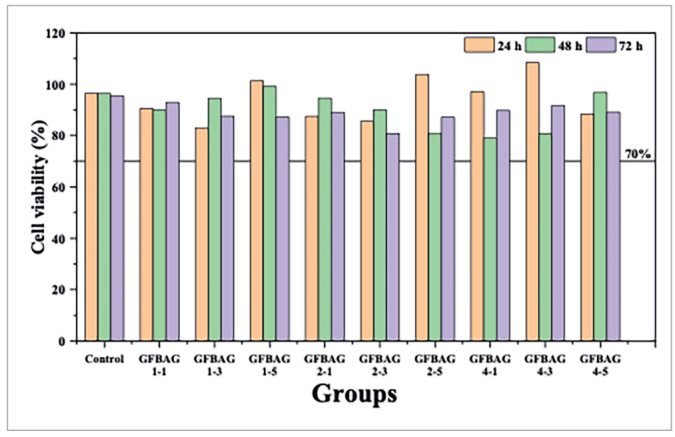
L929 mouse fibroblast cell viability after 24, 48, and 72 h.

Cell viability of the primer extracts was evaluated after 24, 48, and 72 h. The results of the MTT assay (Fig 2) revealed that all the tested primers have cell viability above 70% (ISO standard requirements of acceptable cell viability level), with the lower rate of cell viability observed in GFBAG 1-3 primer after 24 h (83.05%) and in GFBAG 4-1 after 48 h (78.97%). While GFBAG 2-3 primer showed the lowest cell viability rate after 72 h (80.80%). The ANOVA test revealed a significant difference between groups after 24 h (p = 0.000), and the Tuckey HSD tests showed that significant differences were only present between the control group and the GFBAG 1-3 (p = 0.020) and GFBAG 4-3 (p = 0.048) groups. The Welch test was used to compare the groups after 48 and 72 h since there was a concern about data homogeneity. Although the Welch test revealed a significant difference after 48 (p = 0.000) and 72 (p = 0.011) h, the Games–Howell test revealed a significant difference only between the control group and GFBAG 4-3 (p = 0.007) after 48 h with no significant difference between the experimental and the control primer groups after 72 h.

### Shear Bond Strength (SBS)

The descriptive statistics of SBS of all groups are presented in Table 2. All the groups have SBS higher than the control group with the highest value observed in GFBAG 2-3, The ANOVA test revealed a statistically significant difference (p = 0.000) in SBS among groups. The Tukey HSD post hoc test revealed that the significant difference was only between the control groups and GFBAG 1-1, GFBAG 2-3, and GFBAG 2-5 (p = 0.002, 0.000, and 0.009, respectively).

**Table 2:** Descriptive statistics, ANOVA test, and Tukey HSD post hoc multiple comparisons test of SBS for the experimental groups

Group	Descriptive Statistics SBS					Statistics			
	N	Minimum	Maximum	Mean MPa	SD	ANOVA	Tukey HSD Post Hoc Multiple Comparisons		
Control	10	7.13	19.76	12.43	5.09	*Between groups:* *Sum of squares = 2184.95* *df = 9* *Mean square =* * 242.77* *F = 4.68* *p = 0.000*	Group		p
GFBAG 1-1	10	12.83	36.47	26.02	8.74		Control	GFBAG 1-1	**0.002**
GFBAG 1-3	10	10.59	31.99	20.17	8.43			GFBAG 1-3	0.336
GFBAG 1-5	10	11.21	29.54	21.17	6.45			GFBAG 1-5	0.184
GFBAG 2-1	10	13.24	31.17	21.66	6.93			GFBAG 2-1	0.131
GFBAG 2-3	10	18.34	40.34	27.40	6.99			GFBAG 2-3	**0.000**
GFBAG 2-5	10	13.24	34.02	24.69	6.98			GFBAG 2-5	**0.009**
GFBAG 4-1	10	9.78	28.11	15.08	7.14			GFBAG 4-1	0.998
GFBAG 4-3	10	9.58	26.28	15.50	6.41			GFBAG 4-3	0.994
GFBAG 4-5	10	9.78	31.99	19.19	8.12			GFBAG 4-5	0.532

### Adhesive Remnant Index (ARI) and Enamel Damage Index (EDI)

The results of ARI are presented in Table 3. The ARI for most of the experimental groups was higher than the control group. However, a comparison among the groups using the Kruskal–Wallis test showed no statistically significant difference.

**Table 3:** Adhesive remnant index (ARI) and enamel damage index (EDI) of the experimental groups

Group	N	ARI				Median	Interquartile range	Kruskal–Wallis	EDI				Median	Interquartile range	Kruskal–Wallis
		0	1	2	3				0	1	2	3			
Control	10	1	4	4	1	1.5	1	*Test* *statistic = 5.29* *df = 9* *p = 0.808*	4	0	6	0	2	2	*Test* *statistic = 14.12* *df = 9* *p = 0.118*
GFBAG 1-1	10	1	2	5	2	2	1		9	0	1	0	0	0	
GFBAG 1-3	10	2	0	6	2	2	1		7	0	2	1	0	2	
GFBAG 1-5	10	1	1	7	1	2	0		7	0	3	0	0	2	
GFBAG 2-1	10	1	4	4	1	1.5	1		6	1	3	0	0	2	
GFBAG 2-3	10	0	2	7	1	2	0		8	0	2	0	0	1	
GFBAG 2-5	10	0	2	7	1	2	0		3	0	7	0	2	2	
GFBAG 4-1	10	0	2	5	3	2	1		6	1	3	0	0	2	
GFBAG 4-3	10	1	1	5	3	2	1		8	1	1	0	0	0	
GFBAG 4-5	10	3	1	3	3	2	3		7	1	2	0	0	1	

The results of EDI (Table 3) revealed that most of the experimental groups represent lower EDI than the control group except GFBAG 2-5. However, a comparison among the groups using the Kruskal–Wallis test showed no statistically significant difference.

### Antibacterial Test

The results of the inhibition zone against *Streptococcus mutans* are presented in Table 4. All the experimental groups showed higher inhibition zones than the control group. Testing the normality by the Shapiro–Wilk test showed that most of the groups did not violate the assumption of normality (p > 0.05), while the assumption of homogeneity of variance among groups (Levene’s test) was violated (p = 0.011). Therefore, the Welch and the Games–Howell tests were used to analyze the difference in means between the control group and the experimental groups which showed significant differences between all the experimental groups and the control group.

**Table 4:** Descriptive statistics, normality test, Welch test, and Games–Howell multiple comparisons of antibacterial test for the experimental groups

Group	Descriptive Statistics antibacterial test			Shapiro–Wilk			Welch	Multiple Comparisons/Games–Howell		
	N	Mean	SD	Statistic	df	p				
Control	10	3.72	4.81	0.66	10	0.000	*Statistic = 16.41* *df1 = 9* *df2 = 36.55* *p = 0.000*	Group		p
GFBAG 1-1	10	20.91	3.68	0.96	10	0.785		Control	GFBAG 1-1	**0.000**
GFBAG 1-3	10	14.93	2.75	0.85	10	0.059			GFBAG 1-3	**0.000**
GFBAG 1-5	10	13.48	2.65	0.88	10	0.124			GFBAG 1-5	**0.002**
GFBAG 2-1	10	15.16	1.89	0.90	10	0.236			GFBAG 2-1	**0.000**
GFBAG 2-3	10	17.33	3.42	0.94	10	0.599			GFBAG 2-3	**0.000**
GFBAG 2-5	10	18.03	2.97	0.95	10	0.649			GFBAG 2-5	**0.000**
GFBAG 4-1	10	20.28	3.52	0.74	10	0.002			GFBAG 4-1	**0.000**
GFBAG 4-3	10	20.39	2.18	0.95	10	0.661			GFBAG 4-3	**0.000**
GFBAG 4-5	10	19.75	2.27	0.87	10	0.109			GFBAG 4-5	**0.000**

### Evaluation of WSLs Development and Mineral Content of Enamel Surface

#### Laser fluorescence measurements

**Fig 3:**
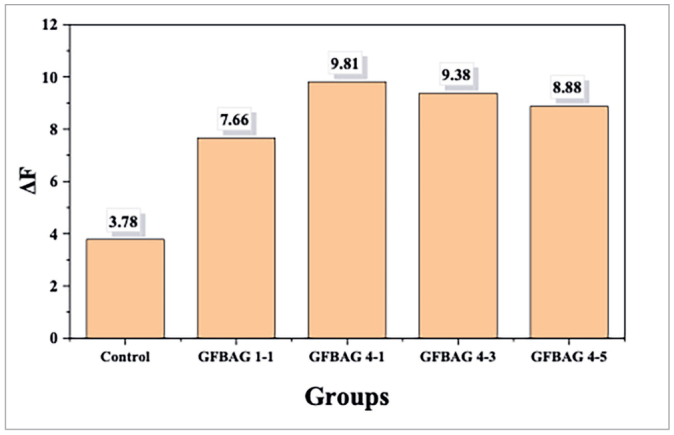
The differences in laser fluorescence measurements (ΔF) by the DIAGNOdent pen after pH cycling.

Based on the previous results, the GFBAG 2-5 group with relatively higher enamel damage value and the groups with comparable lower antibacterial properties (GFBAG 1-3, GFBAG 1-5, GFBAG 2-1, GFBAG 2-3) were excluded from the study. The remineralization properties were evaluated for groups with higher antibacterial properties, and other properties being adequate (GFBAG 1-1, GFBAG 4-1, GFBAG 4-3, GFBAG 4-5). The reduction in the laser fluorescence values (ΔF) was higher in the selected groups than in the control group with no statistically significant difference between them according to the Kruskal–Wallis test (p = 0.122) (Fig 3).

#### Assessment of mineral contents of enamel topography

**Fig 4:**
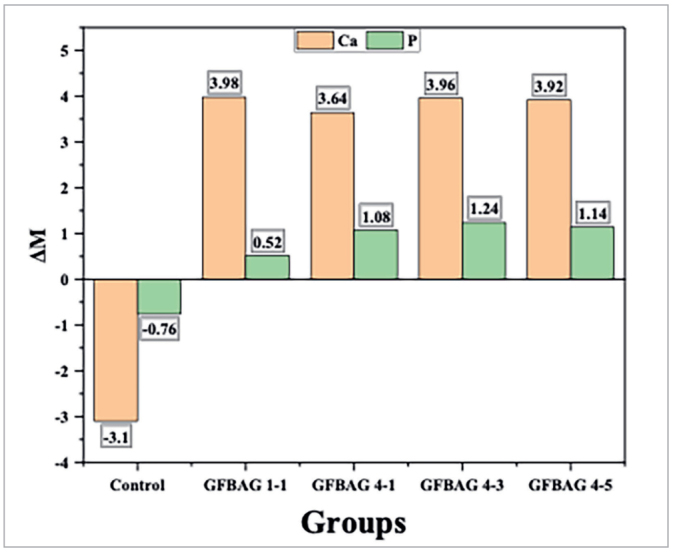
The difference in calcium and phosphorus weight percentage (ΔM) after pH cycling.

The changes in the weight percentage of calcium and phosphorus after pH cycling are presented in Figure 4. Comparison between groups was performed using the ANOVA and Tukey HSD tests (Table 5). All the experimental groups showed statistically significant differences with the control group in the weight percentage of calcium and phosphorus.

**Table 5:** ANOVA test and Tukey HSD for the changes in weight percentage (ΔM) of calcium and phosphorus after pH cycling

ANOVA Test For EDX Result For Ca And P							Multiple Comparisons/Tukey HSD				
Ion		Sum of Squares	df	Mean Square	F	p	Group Name		Mean Difference	SE	p
Ca	Between Groups	194.98	4	48.75	18.82	**0.000**	Control	GFBAG 1-1	–7.08	1.02	**0.000**
	Within Groups	51.80	20	2.59				GFBAG 4-1	–6.74	1.02	**0.000**
	Total	246.78	24					GFBAG 4-3	–7.06	1.02	**0.000**
								GFBAG 4-5	–7.02	1.02	**0.000**
P	Between Groups	13.89	4	3.47	23.37	**0.000**	Control	GFBAG 1-1	–1.28	0.24	**0.000**
	Within Groups	2.97	20	0.15				GFBAG 4-1	–1.84	0.24	**0.000**
	Total	16.86	24					GFBAG 4-3	–2.00	0.24	**0.000**
								GFBAG 4-5	–1.9	0.24	**0.000**

## Discussion

Unfortunately, fixed orthodontic appliances and their accessories act as plaque retentive factors, challenging the maintenance of good oral hygiene and increasing the risk of enamel demineralization and developing WSLs.^24^ The prevention of WSLs can be achieved by the patient training for tooth brushing and using fluoridated mouthwash. Oral hygiene instruction should be the first choice for professionals; however, these methods require patient cooperation, which is usually unpredictable.^48^ The development of an orthodontic adhesive system with the ability to prevent adverse sequelae of orthodontic treatment is the goal of many researchers. This can be achieved by enhancing antibacterial and remineralization properties with a reduction of enamel damage while maintaining adequate bond strength.^54^ The addition of various antibacterial agents to the orthodontic adhesive system has been previously investigated. However, the material properties can be jeopardized, such as chlorhexidine, which has a negative impact on the bond strength.^8^ The present study was designed to investigate the characterization and properties of newly developed orthodontic primers with different concentrations of GF and BAG. The antibacterial properties and the fluoride-releasing ability of GF uprising their biomedical application.^62^ Moreover, the ability of BAG to bind with bone and hard tissue encourages their use in different medical and dental fields.^35^ Additionally, these materials can be retained in etched enamel surfaces rendering them more resistant to the development of WSLs.^69^ The null hypothesis is partially rejected as there are significant differences between the control group and some of the experimental groups in terms of cell viability, bonding strength, antibacterial, and remineralization properties.

Although the used GF and BAG were produced by a specialized manufacturer, the material composition and properties were verified and confirmed through evaluation of their particle shape and size by FESEM, crystal size, and material phase by XRD, and their elemental components by EDX.

Biocompatibility is a necessity of any newly developed materials that are in direct contact with living tissues.^26^ Since GF^66^ and BAG^13^ are biocompatible materials, all the prepared primers in this study showed cell viability levels above 70% indicating convenient biocompatibility according to ISO requirements.^27^

The addition of biomaterials to the orthodontic adhesive system may influence the bond strength of the orthodontic bracket. Maintaining adequate SBS during orthodontic treatment with a safe detachment of fixed appliance components at the end of the treatment is a requirement of a successful adhesive system.^31^ Therefore, in this study, the SBS value was assessed together with the measurement of ARI and EDI. The result of SBS shows that all the experimental groups have higher SBS than the control group. This could be associated with the improvement of the mechanical properties after the addition of GF.^62^ In addition to the possibility of increased mechanical interlocking with the glass degradation and agitate formation on the surface of the primer.^2^

The increase in bond strength was reported in many studies that investigated the addition of BAG to different components of the adhesive system. As Lee et al^39^ who investigated the addition of BAG with silver and zinc to orthodontic primer, Choi et al^17^ who reported an increase in SBS after the addition of BAG to self-adhesive resins, and Song et al^61^ who investigated the effect of the addition of gallium-doped BAG to high-flow orthodontic resin. While controversial results were reported by Park et al^52^ and Nam et al.^48^

The evaluation of ARI results showed no significant differences between the control group and experimental groups with the predominance of scores 1 and 2, indicating that adhesives are partially left on the tooth surface. The failure mode within the adhesive or at the bracket-adhesive interface may have the advantage of avoiding enamel damage and cracks.^53^ This finding came in accordance with the previous studies.^17,35,39^

The process of bracket debonding can produce microcracks and subsequent enamel damage.^21^ Therefore, EDI was recorded after bracket debonding and adhesive removal, the results showed that most of the experimental groups showed lower enamel damage than the control group except for GFBAG 2-5 which showed slightly higher enamel damage values. The reduction in enamel damage could be related to the ability of BAG to exchange Ca and H ions forming Si-OH functional groups on the enamel surface. The Si-OH group could adhere and precipitate on the tooth surface with the ability of hydroxyapatite formation.^40^ Additionally, fluorapatite could be produced by the existence of fluoride ions from GF rendering the enamel surface more resistant to acid etching and subsequent damage after bracket debonding.^62^

The antibacterial properties of BAG and graphene-based materials augment their incorporation as a filler to various dental adhesive systems. The direct contact method was used to examine the antibacterial properties of the developed experimental primers. This method is considered one of the reliable and standard methods for rapid and realistic evaluation of antibacterial properties. All the experimental groups showed an increase in their antibacterial properties when compared with the control group, this could be related to the synergistic antibacterial effect of BAG and GF. The buffering action of BAG with the ability to increase the pH and osmotic pressure was combined with the antibacterial properties of GF which can be attributed to different physical and chemical mechanisms, in addition to the antibacterial effect of the fluoride ion. This result came in accordance with the study by Nam et al^48^ which found an improvement in the antibacterial properties by the addition of fluorinated graphite and BAG to high-flow orthodontic adhesive.

 The four groups (GFBAG 1-1, GFBAG 4-1, GFBAG 4-3, GFBAG 4-5) with the best antibacterial properties, relatively minimal EDI, and adequate SBS were selected for evaluation of the remineralization properties. All the experimental groups showed higher reductions in laser fluorescence than the control group after pH cycling. Although these reductions in laser fluorescence measurement were not statistically significant, they could be of clinical importance. This result was further confirmed by quantitative assessments of enamel mineral content using EDX to evaluate the change in weight percentage of calcium and phosphorus. All the experimental primers showed an increase in calcium and phosphorus values in contrast to the control primer which showed a reduction in the weight percentage after pH cycling. The addition of BAG to orthodontic adhesive resin was found to enhance remineralization by previous studies.^17,40,48,49^ The demineralization could be decreased by saturation of the enamel surface with the released calcium and phosphorus ions from BAG.^37^ Furthermore, the release of ions and subsequent hydroxyapatite formation were enhanced in an acidic environment.^2^

The *in-vitro* studies provide a standardized procedure for the initial evaluation of newly developed materials. Accordingly, this study was designed to test different concentrations of GF and BAG and used different measurement methods to reach the best GF/BAG combination of the novel primer. However, the current study is a short-term study while the duration of orthodontic treatment is relatively long. Additionally, this *in-vitro* study does not exactly mimic the oral environment regarding saliva contamination and subjecting to different occlusal forces. Therefore, further investigations are required to examine SBS with different aging processes.

The addition of GF can produce a slight change in the color of the primer; however, as the primer is usually applied as a smear layer on the tooth surface and covered by the orthodontic adhesive and orthodontic bracket, the color of the primer was not recognized. Additionally, this is a preliminary study that can be followed by an investigation to modify the composition to overcome the color changes of the primer. Therefore, further investigations of the physical and mechanical properties of the newly developed primers, such as hardness, color changes, and degree of conversation, need to be carried out in future studies.

The results could indicate a clinical important difference (albeit not statistically significant) favoring the four selected groups (GFBAG 1-1, GFBAG 4-1, GFBAG 4-3, GFBAG 4-5) in terms of EDI and laser fluorescence measurements. However, this could be more evident with larger sample size.

The recognizable mechanical and biological properties of the GF/BAG-containing primer make it a strong candidate for clinical evaluation in patients with a high risk of WSLs development during orthodontic treatment. The incorporation of antibacterial and remineralizing filler in orthodontic primer rather than adhesive seems to be more logical and beneficial from the clinical approach. This is because the penetration of the etched enamel surface with the low-viscosity primer enhances the complete sealing around the orthodontic bracket, which is the area with a higher risk for WSLs development and strengthens the outer enamel surface against acid attack. Therefore, randomized clinical trials can be designed to assess the effectiveness of the newly developed primer to prevent WSLs development and subsequent bracket failure.

## Conclusions

With the limitations of the current *in-vitro* study, the following can be concluded:

1. All the prepared primers with different concentrations of GF and BAG showed cell viability levels above 70% indicating convenient biocompatibility for clinical application according to ISO requirements.
2. The addition of GF and BAG positively influences the SBS, antibacterial, and remineralizing properties.
3. Most of the prepared primers showed less damage to the tooth structure compared to the unmodified primer after the removal of orthodontic adhesive.
4. The newly developed GFBAG 1-1 and GFBAG 4-3 experimental primers revealed the best results in terms of EDI, antibacterial properties, and calcium deposition.
5. The newly developed GFBAG 4-1 and GFBAG 4-3 experimental primers revealed the best result in terms of reduction in fluorescence measurements.
6. The newly developed GFBAG 4-3 and GFBAG 4-5 experimental primers revealed the best result in terms of phosphorus deposition.

A randomized clinical trial using GFBAG 4-3 experimental primers is suggested to investigate its preventive measure against WSLs development *in-vivo*.
